# Supplementary figures and images for: Genome Size Variation and Comparative Genomics Reveal Intraspecific Diversity in Brassica rapa
Source: Front Plant Sci. 2020 Nov 12;11:577536. doi: 10.3389/fpls.2020.577536 (PMC7689015; doi:10.3389/fpls.2020.577536)

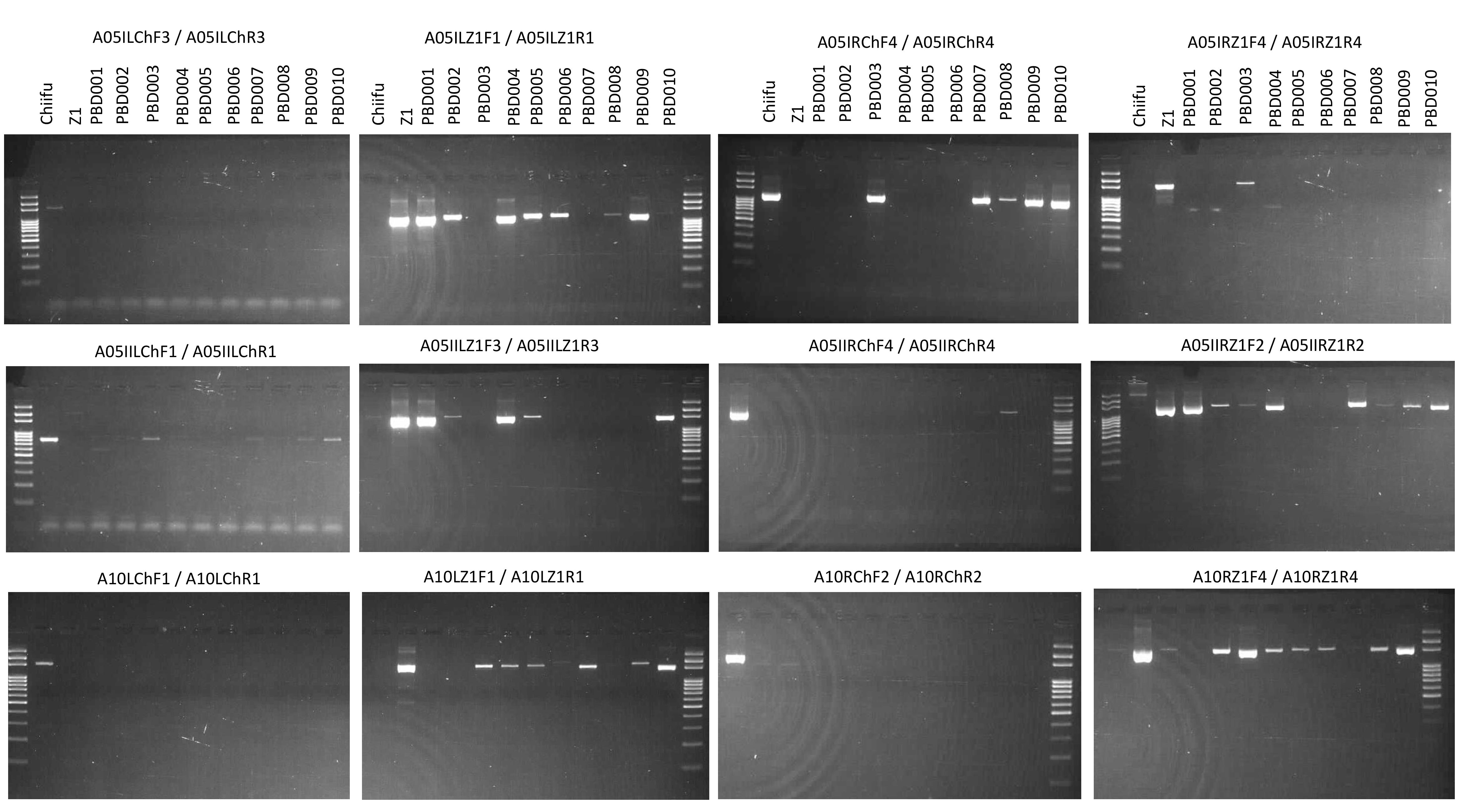

Supplement: Supplementary Figure 1 — PCR amplifications and agarose gels validating inversions in three genomic regions on (A) chromosome A05 first inversion, (B) chromosome A05 second inversion and (C) chromosome A10. Both sides of each inversion have been investigated (L for left side and R for right side) in the focal accessions and B. rapa core collections (named here as PBD1 to PBD10), ordered on the gel as follows: ‘Chiifu,’ ‘Z1,’ PBD001 to PBD010. [file Image_1.JPEG]
